# Acupuncture as an adjunct therapy for enhancing endometrial receptivity in female infertility: a literature review

**DOI:** 10.3389/fphys.2025.1548737

**Published:** 2025-08-07

**Authors:** Shike Zhang, Hui He, Jingyuan Wang, Li Ma, Xiaoyu Wei, Mingxing Zhang, Yi Guo

**Affiliations:** ^1^ Research Center of Experimental Acupuncture, Tianjin University of Traditional Chinese Medicine, Tianjin, China; ^2^ National Clinical Research Center for Chinese Medicine Acupuncture and Moxibustion, Tianjin, China; ^3^ Department of Reproductive Medicine, First Affiliated Hospital of Zhengzhou University, Zhengzhou, China; ^4^ School of Traditional Chinese Medicine, Tianjin University of Traditional Chinese Medicine, Tianjin, China; ^5^ School of Integrated Traditional Chinese and Western Medicine, Tianjin University of Traditional Chinese Medicine, Tianjin, China

**Keywords:** female infertility, endometrial receptivity, acupuncture, endometrial morphology, endometrial blood flow, hormone levels, molecular markers, immune-inflammatory microenvironment

## Abstract

Infertility remains a global challenge, with female factors accounting for the majority of cases. Endometrial receptivity (ER), the ability of the endometrium to accept and support embryo implantation, is a critical determinant of successful conception. Traditional Chinese medicine, specifically acupuncture, has been widely adopted as an adjunct therapy for enhancing ER and improving reproductive outcomes in infertile women. This literature review explores the efficacy and underlying mechanisms of acupuncture in promoting ER by focusing on key areas, including improvements in endometrial morphology, increasing uterine blood flow, adjustment to hormone levels, regulation of molecular markers, modulating endometrial immune-inflammatory microenvironment, and probably activating the somatosensory-autonomic reflex pathway. Although promising, existing studies on acupuncture and ER often face limitations in sample size and methodological rigor, highlighting the need for larger, high-quality randomized controlled trials (RCT). Furthermore, the safety profile of acupuncture in infertility treatment is favorable, with few reported adverse effects. These findings suggest that acupuncture could be a viable complementary therapy for improving pregnancy outcomes in women with compromised ER, although further research is essential to establish definitive protocols and mechanisms.

## Introduction

In recent years, infertility has become a significant global issue, with the incidence showing an increasing trend year by year (Kim and Nho, 2022). According to statistics, female factors account for 70%–80% of infertility cases, and the prevalence among childbearing-aged women is estimated at one in every seven couples in the Western world and one in every four couples in developing countries (Vander Borght and Wyns, 2018). These infertility cases in women can arise from various underlying causes, including issues related to ovulation, fallopian tubes, peritoneal environment, uterus, and cervix (Mattox, 1982).

As the inner lining of the uterus, the endometrium is a crucial reproductive organ that plays a vital role in a woman’s reproductive life and warrants significant attention. The endometrium, is a highly dynamic tissue that reacts to estrogen (E2) and progesterone (P) from the ovaries. These in turn drive the endometrium to multiply, secrete, and exfoliate changes periodically during the menstrual cycle. In the secretory phase, a series of synchronized organizational, morphological, cytochemical, and molecular changes cause the endometrium to enter a receptive state during the so-called “window of implantation (WOI)”, which lasts only 3–6 days in most women and is commonly assumed to begin 6–10 days after ovulation.” During this narrow period of WOI, the endometrium matures and acquires the ability to allow the blastocyst to penetrate and implant in the endometrial stroma and vessels. This ability is referred to as “endometrial receptivity (ER)” (Les et al., 2019). Successful implantation is a complex and demanding process that requires synchronized communication between a healthy embryo and a receptive endometrium (Diedrich et al., 2007; Governini et al., 2021). If, for any reason, this process fails to establish or is disturbed, the implantation is unsuccessful.

In recent decades, advancements in reproductive medicine have led to fed to the development of assisted reproductive technology (ART), such as *in vitro* fertilization/intracytoplasmic sperm injection-embryo transfer (IVF/ICSI-ET), which has significantly helped numerous couples overcome infertility challenges. However, despite these advancements, the average implantation success rate in IVF-ET remains relatively low, at around 25% (De los Santos et al., 2003). While improvement in laboratory and *in vitro* culture conditions have enhanced the selection of top-quality embryos, they still do not guarantee satisfactory reproductive outcomes (Szamatowicz, 2022; Polanski and Baumgarten, 2021; Szamatowicz, 2016). Recurrent implantation failure (RIF) is a devastating reality for women undergoing IVF and a major challenge for clinicians (Shaulov et al., 2020). It is estimated that among implantation failures, poor quality embryos account for one-third, while the remaining two-thirds are attributed to defective ER and impaired embryo-endometrial dialogue (Craciunas et al., 2019; Idelevich and Vilella, 2020). In addition, defective ER not only leads to infertility and/or pregnancy loss, but also has an increased risk of pregnancy complications even if conception occurs, such as preeclampsia, intrauterine growth restriction and/or stillbirth (Neykova et al., 2022; Revel, 2012). In contrary, enhanced ER has demonstrated positive effects on embryo implantation rate (EIR), clinical pregnancy rate (CPR) and live birth rate (LBR) (Koo et al., 2021; Kay et al., 2021; Liu et al., 2019). Consequently, improving ER is a critical intervention for treating female infertility, promoting embryo implantation, and increasing pregnancy rates in IVF.

Currently, western medical interventions commonly used to improve ER include E2 and P (Paulson, 2011), growth hormone (Altmäe and Aghajanova, 2019), human chorionic gonadotropin (HCG) (Makrigiannakis et al., 2017), platelet-rich plasma (Mouanness et al., 2021) sildenafil citrate (Hajipour et al., 2021), endometrial injury (Sar-Shalom Nahshon et al., 2019), and surgical treatments (Evans-Hoeker and Young, 2014). These approaches aim to increase the endometrial thickness (EMT), enhance endometrial morphology, and improve uterine microcirculation, thereby achieving favorable clinical outcomes. However, there is no consensus on the optimal timing, dosage, or administration routes for ovarian stimulation medications. Additionally, excessive use of these drugs may have adverse effects on ER, the embryo, and implantation (Liang et al., 2018; Santos et al., 2010). Therefore, there is a need to develop safer and more effective interventions.

Acupuncture is a key therapy in traditional Chinese medicine (TCM), with a history spanning over 2000 years (Zhou and Benharash, 2014). As non-pharmacological remedy with considerable efficacy, acupuncture has become increasingly popular in infertility treatment. A survey conducted in the United Kingdom reported that acupuncture use among female infertile patients has increased fivefold in the past 10 years (Hopton et al., 2012). This increase is parallel to the growing evidence that acupuncture combined with embryo transfer can increase CPR in women undergoing IVF-ET (Wang et al., 2021). Acupuncture treatment has also been proved to produce a promising efficacy in improving poor ER in infertile patients. A recent systematic review and meta-analysis has suggested that acupuncture may improve the pregnancy rate, embryo transfer rate, increase trilinear endometrium, thicken endometrium, reduce resistive index (RI), pulsatility index (PI), and peak systolic velocity/end-diastolic blood velocity (S/D), compared with medication, sham acupuncture or physiotherapy. Studies indicated that acupuncture may influence ER via various mechanisms (Zhong et al., 2019). For example, Xing et al. showed that acupuncture could improve ER-related factors including endometrial morphology, pinopodes, homeoboxA-10 (HOXA10), and leukemia inhibitory factor (LIF) protein expression, as well as angiogenesis by activating the PI3K/AKT pathway, thus promoting ER and the number of implantation sites (Xing et al., 2022). Aadditionally, high-frequency electroacupuncture (EA) could improve ER by reducing the protein expression levels of E-cadherin, β-catenin, and claudin-1 (CLDN1) adhesion molecules, and activating the LIF/STAT3 signaling pathway (You et al., 2021). Although the precise molecular mechanisms by which acupuncture improves ER are still unclear, it is indeed widely used by clinicians to enhance ER, and acupuncture is a safe treatment with relatively fewer side effects. Up to now, reviews that summarize the effects of acupuncture on ER both in clinical patients and animal models are rare. Against this backdrop, the present study aims to review the efficacy and mechanism of acupuncture for enhancing ER, with the goal of providing evidence to further improve clinical outcomes for infertile patients with suboptimal ER.

### Endometrial receptivity assessment

To date, various modalities, including endometrial biopsy, ultrasound, endometrial receptivity array (ERA) tests, endometrial fluid aspiration, and hysteroscopy, have been developed to assess ER. Numerous markers have been identified as being closely associated with ER (Craciunas et al., 2019). Among the earliest methods, endometrial histological dating based on Noyes’ criteria provided a classic approach to evaluate the WOI by defining various morphological stages of endometrial development, including menstrual, proliferative, and secretory phases. Notably, the mid-secretory phase corresponds to the WOI. However, recent studies have suggested that this histologic evaluation was somewhat outdated, while novel and updated methods have been proposed to as replacements for this traditional standard. Pinopodes have been identified as well-known ultrastructural markers of ER, as their abundance and morphology align closely with the timing of WOI. In clinical practice, ultrasonography is the preferred method for evaluating endometrial morphology, due to its non-invasive nature and availability. Ultrasound is commonly used to measure various endometrial characteristics, including EMT, volume, pattern, Doppler signals, and wave-like activity of the endometrium. Among them, EMT is the most frequently investigated marker of ER. However, ultrasound measurements have limited predictive value for clinical pregnancy, preventing their use reliable diagnostic tools for ER. With advances in medical research, the ERA has emerged as a promising molecular instrument for diagnosing ER. ERA is a microarray technology that characterizes the transcriptomic signature of human ER by analysing 238 differentially expressed genes during WOI, allowing for the prediction of ER timing and WOI, and guiding personalized embryo transfer for infertile patients. Although numerous studies have supported the clinical value of the ERA test, some negative results still remain, and further large prospective studies are needed to fully assess its efficacy. This ongoing uncertainty underscores the challenge of diagnosing ER accurately, due to the lack of a reliable, non-invasive, and clinically applicable test.

### Factors of defective endometrial receptivity

The contributing factors that cause defective ER may not yet be fully recognized, but the causes are multifactorial and include endocrine disorders (e.g., polycystic ovary syndrome (PCOS)), thin endometrium, uterine anomalies (e.g., leiomyomas, polyps, adenomyosis, septa), chronic endometritis (CE), and immunologically mediated disturbances. In the following sections, we will explore each of these factors in detail to better understand their roles in ER defects.

## PCOS

As one of the most common endocrine disorders, PCOS affects 7%–10% of women of childbearing-age and often leads to reproductive dysfunction (Bozdag et al., 2016). Numerous studies have identified disturbances in hormone levels and metabolic profiles, such as obesity, glucose metabolism issues, hyperinsulinemia, and hyperandrogenism. These factors collectively create a hostile effect on ER during WOI in women with PCOS, resulting in an unfavorable uterine microenvironment for embryo implantation (Pathare et al., 2022). Nowadays, numerous molecular indicators closely related to ER have been found to exhibit significant alterations in patients with PCOS, including HOXA, LIF, pinopodes, integrin (ITG) αvβ3, and intercellular junctions (Jiang and Li, 2022). Impaired ER, leading to recurrent abortion and implantation failure, can contribute to subfertility in women with PCOS. In terms of treatment, letrozole, the most commonly used ovulation-stimulating drugs for PCOS, has been shown to be more effective than clomiphene in improving ER and increasing pregnancy rates (Wang et al., 2019). In addition, metformin may enhance ER by upregulating the expression of HOXA10 and ITGB3 through downregulating the expression of miR-491-3p and miR-1910-3p in the endometrium of women with PCOS undergoing IVF/ICSI-ET (Zhai et al., 2019).

### Thin endometrium

EMT is the most classic and widely used indicator for evaluating ER, and adequate EMT at the time of WOI is a requisite for successful implantation (Kunicki et al., 2014). In 2018, the expert consensus of the Reproductive Medicine Branch of the Chinese Medical Association (CSRM) defined a thin endometrium as an EMT of less than <7 mm on the day of HCG administration, as measured by ultrasound. Highlighting the underlying mechanisms, Miwa et al. demonstrated that the pathophysiologic features of a “thin” endometrium were characterized by high blood flow impedance of uterine radial artery, impaired epithelial growth, decreased vascular endothelial growth factor (VEGF) expression, and poor vascular development (Kasius et al., 2014). Numerous studies have reported a close association between thin endometrium and adverse reproductive outcomes. A recent systematic review and meta-analysis involving 22 studies found that EMT of ≤7 mm was associated with significantly lower implantation rate compared to cases with EMT >7 mm [23.3% versus 48.1%, OR 0.42 (95% CI 0.27–0.67)] (Miwa et al., 2009). Furthermore, EMT ≤7.5 mm (or <8 mm) was independently associated with a higher risk of low birthweight, preterm birth, and miscarriageamong women undergoing frozen IVF/ICSI-ET with singleton newborns (He et al., 2022; Zheng et al., 2022a). Cycle cancellation or postponement until adequate endometrial development is therefore recommended for affected patients. When EMT is ≥9–10 mm, the CPR can significantly increase (Kovacs et al., 2003).

### Uterine anomalies

Polyps, adenomyosis, and leiomyomas are common uterine anomalies in clinic among women with or without fertility (Munro, 2019). Endometrial polyps are localized endothelial tumors composed of endometrial glands, stroma, blood vessels, and fibrous tissues. Adenomyosis is a condition in which normal endometrial glands and stroma grow into the myometrium, forming a diffuse or focal lesion. Leiomyomas, also called “fibroids” or “myomas”, are thought to be benign monoclonal growths that emerge from uterine smooth muscle cells and fibroblasts. Present studies suggest that these entities may contribute to female infertility by impairing ER (Munro, 2019; Rackow et al., 2011; Sunkara and Khan, 2012). Indeed, a substantial body of evidence has shown that polyps, adenomyosis, and leiomyomas have all been associated with an increased likelihood of abnormal endometrial molecular expressions, especially HOXA-10 and LIF (Fischer et al., 2011; Xiao et al., 2010; Makker et al., 2017), which is thought to have deleterious effect on implantation and early embryo development, thus resulting in lower pregnancy rate (Vercellini et al., 2014).

### Chronic endometritis

CE is a chronic and mild local inflammatory disease manifested by aberrant plasmacyte infiltration into the endometrial stroma. The prevalence of CE is estimated to be as high as 15%–57.5% among women affected by infertility, IVF implantation failure, and unexplained recurrent miscarriage (Zeng et al., 2022). Although CE patients often lack typical clinical symptoms, their chronic inflammatory microenvironment can affect reproductive function through multiple mechanisms: firstly, the inflammation within the endometrium may cause cellular and biochemical changes that disrupt the individual WOI, leading to embryo-endometrium asynchrony (Kuroda et al., 2020); Secondly, the decreased ER in CE is characterized by reduction in mature pinopodes and low expression of LIF in the surface epithelium (Kogan et al., 2012); In addition, protein and mRNA levels of hypoxia-inducible factor 1α (HIF1α), vascular endothelial growth factor A (VEGFA), vascular endothelial growth factor receptor 2 (VEGFR2), as well as microvascular density (MVD) were significantly upregulated in women with CE, offering a new perspective on reduced ER in CE-associated infertility (Liu et al., 2022). However, when the endometrial inflammatory state is relieved through effective antibiotic treatment, reproductive outcomes can be improved, with increased pregnancy and live birth rates in patients with unexplained recurrent pregnancy loss (RPL) and enhanced ongoing pregnancy rate in those with RIF (Puente et al., 2020).

## Immunologically mediated disturbances

The endometrium serves as a critical interface for maternal-fetal immunological communication, with emerging evidence implicating uterine natural killer (uNK) cells and regulatory T cells (Tregs) in both endometrial tissue and peripheral blood as key mediators of implantation success (Ticconi et al., 2019; Kofod et al., 2018). These immunologic changes in peripheral blood and the uterus can, in turn, affect embryo implantation. Clinical investigations reveal that during frozen embryo transfer (FET) cycles, viable pregnancies demonstrate significantly elevated endometrial and peripheral blood Treg populations alongside reduced CD16^+^ uNK subsets compared to non-pregnant controls (Sauerbrun-Cutler et al., 2021). In a prospective study, Lédée N, et al. reported that endometrial immune profiles impaired the implantation process and appeared to be dysregulated in 81.7% of the RIF patients compared to control (Lédée et al., 2016). Notably, women with RIF have different kinds of immune dysfunction, characterized by T helper (Th) 1 to Th2 ratio, variations in NK cell and macrophage numbers (Nupur et al., 2023) further suggested. These findings establish RIF as a heterogeneous immune disorder and underscore the necessity for standardized immunological assessment protocols in clinical management (Nupur et al., 2023).

### An in-depth review of Acupuncture’s role in treating endometrial receptivity in female infertility

Acupuncture has been utilized in China for centuries to regulate the female reproductive system. Historical records indicate its use for treating female infertility dates back to the Western Jin Dynasty nearly 1,800 years ago, as documented in the *A-B Classic of Acupuncture and Moxibustion* (*Zhen Jiu Jia Yi Jing*) by the renowned ancient TCM physician Huang Fumi. The first modern study reporting acupuncture as a non-pharmacological therapy for female infertility appeared in 1988, demonstrating comparable efficacy between auricular acupuncture and drug-based therapy in achieving pregnancy (Gerhard and Postneek, 1988). In 2019, researchers led by Xi et al. at Nanjing University of Chinese Medicine in China conducted a comprehensive review of Chinese Medical Classics (5th Edition) to compile a reference to infertility treatments documented before the founding of the People’s Republic of China in 1949 (Jin et al., 2019). They identified 301 clauses detailing acupuncture and moxibustion treatments for infertility, referencing 41 points across 358 points. Frequently cited and prescribed acupoints as a basic prescription for acupuncture included Zhongji (RN3), Guanyuan (RN4), Rangu (KI2), Yongquan (KI1), Yinlian (LR11), Yinjiao (RN7), Shangqiu (SP5), Shiguan (KI18), Qixue (KI13), and Shangliao (BL31), often in combination with moxibustion and syndrome differentiation. Given the rising incidence of low ER among female infertility patients, researches on acupuncture’s role in enhancing ER continue to grow. By analyzing 36 RCTs and summarized the regularities and characteristics of the literature on acupuncture in improving ER, Zhuang et al. showed acupuncture could improve ER by many ways, among which, filiform needle acupuncture, EA and transcutaneous electrical acupoint stimulation (TEAS) have the top three frequencies of use (Zhuang et al., 2019). Furthermore, they revealed six acupoints used more than 10 times, including Zigong (EX-CA1), Guanyuan (RN4), Sanyinjiao (SP6), Zhongji (RN3), Zusanli (ST36), and Qihai (RN6). In 2019, a systematic review included 13 RCTs of women with infertility due to low ER, and found acupuncture may improve pregnancy rate and embryo transfer rate, although very low to moderate level of evidence (Zhong et al., 2019). In 2021, a systematic review suggested that acupuncture therapy could improve the CPR, biochemical pregnancy rate, EIR, and EMT when compared with the control group in patients with RIF, although the quality of the studies was not high (Li et al., 2021). Frequency is a critical parameter in acupuncture stimulation. Extensive researches have reported the therapeutic effects of acupuncture at different frequency settings. In 2022, the systematic review conducted by Zheng et al. suggested the trend of relatively more acupuncture dosage showed better effects for poor ER among infertile women (Zheng et al., 2022b). Additionally, several studies have examined the safety of acupuncture in female infertility. Most importantly, acupuncture has relatively fewer side effects on improvement of ER and is comparatively safe (Zhong et al., 2019). Although the precise mechanisms by which acupuncture improves ER remain unclear, accumulating evidence points to its benefical effects, which warrant further in-depth investigation by many studies. The following section into the efficacy and mechanisms of acupuncture in treating endometrial receptivity in female infertility.

## Efficacy and mechanisms of acupuncture in improving endometrial receptivity among infertile women

### Search strategy and selection critiera

A comprehensive search was conducted across multiple databases including PubMed, Web of Science, Embase, Cochrane Library and Scopus databasesd for relevant studies from inception through December 2024. There was no limitation in country, but only studies published in English were included. The search encompassed clinical trials, animal experiments, systematic reviews, and meta-analyses to ensure a thorough collection of literature related to the topic. We used “acupuncture”, “electroacupuncture”, “transcutaneous electrical acupoint stimulation”, “endometrium”, “endometrial receptivity”, “uterus” and “uterine receptivity”, related terms as key words in our search. In addition, to minimize publication bias, we reviewed reference lists of relevant studies.

### Clinical efficacy of acupuncture in promoting pregnancy outcomes by improving endometrial receptivity

Presently, acupuncture is mainly used as an adjuvant treatment in female infertility, such as PCOS and IVF-ET. Multiple clinical studies have been conducted to provide evidence-based information about the efficacy of acupuncture, EA and TEAS on ER to improve female fertility; the primary reproductive outcomes were ovulation rate, EIR, CPR, and LBR.

In patients with ovulatory disorder infertility, numerous studies indicated the combined intervention of acupuncture with clomiphene or letrozole showed improved effects on the quality of follicle and the receptivity of endometrium, so as to improve the pregnancy rate, which was better than the simple application of medication (Yu et al., 2018; Zheng et al., 2018; Zheng et al., 2019; Yang et al., 2020; Budihastuti et al., 2021). As regards to the effect of acupuncture on endometrium, we found all these studies reported a thicker EMT after acupuncture treatment. Two of them indicated acupuncture improved the endometrial morphology (mainly A-type rate) (Yu et al., 2018; Zheng et al., 2019). Two studies reported acupuncture reduced uterine arterial PI and RI, improved uterine arterial flow (Yang et al., 2020; Budihastuti et al., 2021). Acupuncture has been shown to remarkably increased the E2 and P levels (Yu et al., 2018). In addition, Yang et al. demonstrated acupuncture could enhance the serum HOXA10 expression, thus improving the endometrial receptivity (Yang et al., 2020).

Similarly, in patients with anovulatory infertility undergoing IVF-ET or FET,also revealed that acupuncture could enhance the EIR and CPR by improving the ER (Xu et al., 2022; Wu et al., 2022). In clinical practice, acupuncture and moxibustion also often plays a role by combining other treatments, such as acupuncture combined with gonadotropin-releasing hormone agonists could improve the ER, thereby increasing CPR and improving pregnancy outcomes in patients with RIF (Yang et al., 2024). A study demonstrated in IVF-ET treatment, acupuncture combined with moxibustion affected E2 level on hCG day, improved high-quality embryo rate, endometrial blood flow state and morphology, thus increasing EIR (Chen and Hau, 2015). Meanwhile, the positive effects of acupuncture and moxibustion on ER and pregnancy outcomes in RIF patients were further confirmed by another clinical study (Lan et al., 2023). Several studies, based on TCM syndrome differentiation, suggested that acupuncture combined with Chinese materia medica could reduce the scores of TCM syndrome and improve the pregnancy outcomes in IVF-ET or RIF patients (Sun et al., 2012; You et al., 2023). In infertile patients with thin endometrium type, accumulating studies reported that acupuncture could increase EMT, enhance ER, thus improving the pregnancy outcomes (Xing et al., 2023; Qi et al., 2022a; Xue et al., 2021). In addition, the EA has also shown certain benefits on ER and IVF-ET reproductive outcomes in patients with diminished ovarian reserve (DOR) (Shen et al., 2022).

TEAS is a noninvasive intervention derived from EA and as an alternative to manual or EA. A RCT revealed that TEAS intervention may have beneficial effects on endometrial HOXA10 expression and ultrasound parameters of ER, which may explain the improvement in pregnancy outcomes of patients undergoing FET (Shuai et al., 2015). In patients with poor ovarian response (POR) who were undergoing IVF/ICSI-ET cycles, Qi et al. found adjuvant TEAS on the basis of coenzyme Q10, could significantly improve ovarian responses, increase the numbers of retrieved oocytes and excellent embryos, and improve ER (Qi et al., 2022b). When the stimulus intensity was at 40 mA and above, TEAS could be helpful to improve the patient’s endometrial condition and retrieve more oocytes (Zhai et al., 2022). In addition, TEAS has been proven to significantly improve the IVF-ET pregnancy outcomes in older women over 35 years (Feng et al., 2022).

However, there were some results of studies did not support that acupuncture improve the clinical pregnancy outcomes of IVF (Ho et al., 2009; Dong et al., 2024; Lai et al., 2024; Zhou et al., 2025). What’s more, a study conducted by the University of Hong Kong reached a contrary conclusion, they suggested the placebo acupuncture had significantly higher overall pregnancy rate than the real acupuncture in IVF treatment (So et al., 2009). Interesting, all these studies suggested acupuncture could be helpful to improve the patient’s endometrial conditions. The detailed clinical data on acupuncture for improving pregnancy outcomes and enhancing endometrial receptivity are presented in Table 1.

**TABLE 1:** The clinical studies of acupuncture for improving pregnancy outcomes and enhancing endometrial receptivity.

Year	Author	Subject	Mode	Acupoints	Treatment duration	Significant indicators	
						Pregnancy outcomes	ER-related factors
2018	Yu	PCOS	EA	CV6, CV4, EX-CA1, KI12, SP6, CV3, SP8, BL23, BL22, BL32	3 per wk for 3 months	↑ Ovulation rate	↑ E_2_, P, EMT, A-type rate
2018	Zheng	Ovulatory disorder	A	Group 1: ST25, RN6, RN4, ST29, DU20, EX-HN3, LI4, LI11, ST36, SP6, KI3, LR3; Group 2: BL15, BL17, BL23, BL52, BL32, BL28, DU20, SP6, KI3, alternately used	Alternate days for 2 months	↑ CPR, follicular diameter	↑ EMT
2019	Zheng	Ovulation failure	A	RN12, RN10, RN6, RN4, KI13, ST28, ST29	Every third day to ovulation, for 3 menstrual cycles	↑ Menstrual condition	↑ EMT, endometrial morphology
2020	Yang	PCOS	A	CV4, CV6, ST36, SP6, EX-CA1	Alternate days for 3 menstrual cycles	↑ CPR	↑ EMT, HOXA10 ↑ PI, RI, S/D
2021	Budihastuti	PCOS	EA	CV3, CV6, ST29, SP6, LI4, ST36	12 times	↑ Folliculogenesis	↑ EMT ↓ RI, PI
2022	Xu	FET	A	GV20, GV4, BL17, CV4, CV6	Alternate days until 1 day before ET	↑ EIR, CPR	↑ EMT, endometrial morphology
2022	Wu	IVF-ET	A	Group 1: CV 4, CV6, CV 3, EX-CA1, Group 2: GV4, GV3, BL23 and BL32, alternately used	Alternate days until ET day	↑CPR	↑ Type A rate ↑ serum E2 and P on HCG day
2024	Yang	RIF	A	RN4, RN6, RN3, KI12, BL23, ST36, SP6, KI3, LR3	2 per wk, from GnRHa injection to ET day	↑ EIR ↑ CPR	↑ EMT, type A+ B rate ↑ type III endometrial blood flow ↓ Type I endometrial blood flow
2015	Chen	IVF-ET	A	CV3, CV4, CV6, EX-CA1 SP10	Per day from ovulatory induction till ET day	↑ High-quality embryo rate	↑ Type A rate, serum E2 and P on HCG day ↓ S/D, RI and PI
2023	Lan	RIF	A	GV14, BL18, BL23, CV12, CV6, CV4	3 per wk till the ET day	↑ CPR	↑ EMT, type A rate ↓ PI, RI
2012	Sun	IVF-ET	A	CV3, LR3, EX-CA1, SP6	alternate days from Gn till HCG day	↑ High quality oocyte rate, high quality embryos rate, CPR	↑ Type A rate ↓ RI
2023	You	RIF	A	Group 1: GV20, CV12, CV6, CV4, EX-CA1, Luanchao, SP10, SP6, SP8, KI3, PC6, LR3; Group 2: GV20, BL23, BL18, EX-B7, BL32, alternately used	3 per wk for 3 menstrual cycles	↑ EIR, CPR, LBR	↓ SerumD-dimer, HCY, TXB2, PI, RI
2023	Xing	RIF	A	PC6, SP4+ LI4, SP6, BL18, KI16, EX-CA1, ST28, ST29, DU9, BL23	3 per wk for 3 menstrual cycles	↑ CPR ↑ LBR	↑ EMT, type A + B rate
2022	Qi	FET	A	LR3, SP6, RN4, SP10, ST36	Alternate days from menstruation to ET day	↑ EIR, CPR	↑ EMT, type A rate, HOXA10 AMPK/mTOR pathway ↓ PI, RI and S/D
2021	Xue	RIF	A	GV20, GV14, CV6, CV4	Alternative days for 3 menstrual cycles	↑ CPR	↑ EMT, type A rate ↓ PI and RI
2022	Shen	IVF-ET	EA	BL17, BL23, GV4, EX-B8, BL32, BL33, ST25, CV6, CV4	Alternative days for 3 menstrual cycles	↑ HCG positive rate, EIR, CPR, LBR	↑ Type A rate
2015	Shuai	FET	TEAS	CV3, CV4 SP6, EX-CA1	Alternative days for 3 menstrual cycles	↑ EIR, CPR, LBR	↑ Triple-line pattern rate, HOXA10, endometrial/subendometrial VI
2022	Qi	IVF/ICSI-ET	TEAS	ST25, EX-CA1, RN4, RN, ST36, SP6	Alternative days for 2 menstrual cycles until HCG day	↑ Numbers of MII eggs, excellent embryos	↑ EMT
2022	Zhai	IVF-ET	TEAS	RN4, RN3, SP6, EX-CA1 KI13	Per day for 10–13 days	↑ Number of ova captured	↑ EMT
2022	Feng	IVF-ET	TEAS	SP8, ST29, NR19, SP10; ST36, KI3, BL23, RN4, NR12	Before and after ET	↑ EIR, CPR	↑ Pinopodes, ITGα1β1/αVβ3, LIF, serum P
2009	Ho	IVF-ET	EA	LR3, SP6, ST28, EX-CA1, RN6, RN4	2 per wk for 2 weeks	NS	↓ PI
2024	Dong	IVF-ET	A	CV3, CV6, SP6, LR3, ST25, ST29, ST36	For totally 4 days	NS	↑ VI, FI and VFI
2024	Lai	RIF	EA	GV24, GV4, GB13, EX-CA1, KI16, SP6, SP10	30 min	NS	↑ Type III endometrial blood flow and VI^MV^ for the endometrium and ovary
2025	Zhou	IVF-ET	A	CV4, CV6, CV3, EX-CA1, SP6, LI4, BL23, DU4, BL32, SP10	2–3 per wk till ET for 3 menstrual cycles	NS	↑ Serum E2, EMT ↓ RI; PI
2009	So	IVF-ET	A	PC6, SP8, LR3, GV20, ST36, SP6, SP10, LI4	ET day	Opposite result	↓ Endometrial/subendometrial vascularity

ER, endometrial receptivity; PCOS, polycystic ovary syndrome; EA, electropuncture; wk, Week; E2, estradiol; P, progesterone; EMT, endometrial thickness; A, acupuncture; CPR, clinical pregnancy rate; HOXA10, Homeobox A10; PI, pulsatility index; RI, resistance index; S/D, Peak systolic velocity/diastolic velocity; FET, Frozen-thawed embryo transfer; ET, embryo transplantation; EIR, embryo implantation rate; IVF-ET, Vitro fertilization-embryo transfer; HCG, human chorionic gonadotropin; RIF, recurrent implantation failure; GnRHa, Gonadotropin-releasing hormone agonists; Gn, Gonadotropin; LBR, live birth rate; HCY, homocysteine; TXB2, Thromboxane B2; AMPK, adenosine monophosphate activated protein kinase; mTOR, mammalian target of rapamycin; TEAS, transcutaneous electrical acupoint stimulation; VI, vascularisation index; ICSI, intracytoplasmic sperm injection; MII, Metaphase II; ITG, integrin; LIF, leukemia inhibitory factor; NS, not significant; FI, flow index; VFI, vascularization flow index; VI^MV^, vascular distribution index.

### Animal studies of acupuncture affecting endometrium

Increasing animal experiments have been done to investigate the effect of acupuncture on the endometrium in rats, so as to explore the possible mechanisms underlying improvement in pregnancy outcomes. Several studies have focused on the role of acupuncture in PCOS rats. Previously, acupuncture could conspicuously downregulate the serum levels of T and E2, improve the ovaries and uterus development, promote ovulation, enhance EMT, and advance blastocyte implantation in rats with PCOS (Zhang et al., 2009). Similarly, in the clomiphene-induced rat model, Fu et al. demonstrated the acupuncture group significantly decreased serum E2 levels, and enhanced the glandular area and endometrial LIF and osteopontin (OPN) expression than the control group, while there were no differences in serum P levels, EMT, and stromal area between two groups (Fu et al., 2011). Mechanistically, EA intervention significantly improved the number of blastocyst implantation and pregnancy rate in PCOS rats by up-regulating the mRNA and protein expressions of insulin receptor substrate 1 (IRS1) and insulin receptor substrate 2 (IRS2) in the endometrium (Lai et al., 2016a; Lai et al., 2016b). Additional pathways involve notably increased ER factors including endometrial morphology, pinopodes, HOXA10 and LIF protein expressions, as well as improved endometrial angiogenesis by activating PI3K/AKT pathway, thus promoted implantation sites’ number (Xing et al., 2022).

The effect of acupuncture in repairing thin endometrium have been widely investigated. EA enhances the ER of thin endometrium model rats by improving multiple pinopode-related moleculars, such as ITGαvβ3, HOXA10, heparin-binding EGF-like growth factor (HBEGF), estrogen receptor alpha (ERα), and progesterone receptor (PR) (Xi et al., 2021). Synergistic effects on thin endometrium occur when combined with bone marrow mesenchymal stem cells (BMMSCs), potentially through ER/PR protein upregulation (Meng et al., 2021), or SDF-1/CXCR4 signaling modulation (Xia et al., 2019; Hao et al., 2023).

During the process of controlled ovarian hyperstimulation (COH), large dose of ovulation-inducing drugs can impair the coordinated development of endometrium and embryo, resulting in lower EIR. Acupuncture has been proven to be useful in promoting embryo implantation in COH rats through multiple pathways. Based on the optimal intervention timing,EA intervention during HCG injection day in IVF-ET cycles significantly enhances the insulin-like growth factor 1 (IGF-1) mRNA expression in endometrium, thereby increasing the mean imbed site number, and improve the EIR and CPR (Zhu et al., 2016). In terms of angiogenesis regulation, EA may help facilitate the endometrial angiogenesis through regulating the number and roles of uterus dendritic cells or activating the VEGFR2/PI3K/AKT and VEGFR2/ERK signaling pathways to aid embryo implantation (Dong et al., 2017; Chen et al., 2019). In superovulation mice, acupuncture combined with TCM could increase endometrial pinopodes, EMT, and upregulate the HOXA10 expression by activating PI3K/Akt/mTOR pathway and inhibiting the expression of miR-494-3p, thus increasing the number of blastocysts (Yuan et al., 2020). EA, especially high-frequency EA could effectively improve ER and promote the blastocyst implantation by reducing cell adhesion molecules and enhancing the miRNA-223-3p-mediated LIF/STAT3 signaling pathway (You et al., 2021; You et al., 2022). Acupuncture helps restore the balance of E2 and P levels and the forward shift of the WOI in COH rats (Hu et al., 2023). Furthermore, Zhu et al. firstly reported that EA preconditioning could improve the ER by regulating the expression of clock gene Bmal1 in the uterine tissue, which could restore the balance of clock gene rhythm (Zhu et al., 2024).

Several studies used mifepristone induced “embryo implantation failure model” rats to evaluate the effect of acupuncture in improving the implantation rate. A former research reported acupuncture could increase the weight of the uterus and ovary, improve the development of the endometrium, thus reverse mifepristone’s anti-implantation effect (Liu et al., 2007). Acupuncture treatnt upregulates the expression of CXCL8 receptors (CXCR1 and CXCR2) and CCL2, and increases the subset of uNK cells at maternal-fetal interface were higher (Gao et al., 2014; Gao et al., 2013). Alternatively, acupuncture could improve the poor receptive state of endometrium by increasing LIF and IL-12 secretion, and improving Th2 cytokines secretion, but inhibiting Th1 cytokines to maintain a Th2 dominant environment, thus to promote blastocyst implantation (Gui et al., 2012a; Gui et al., 2012b). The comprehensive information regarding acupuncture in animal research is described in Table 2.

**TABLE 2:** The animal researches regarding effect of acupuncture on the endometrium.

Year	Author	Subject	Mode	Acupoints	Treatment duration	Significant indicators	
						Pregnancy outcomes	ER-related factors
2009	Zhang	PCOS rats	A	CV4, CV3, SP6, CX-CA1	For 5 continuous days	↑ NBI	↑ EMT ↓ serum T, E2
2011	Fu	CC-induced rats	A	SP6, ST36, LR3, CV4, CV3	Per day for 6 days	NR	↑ LIF and OPN, glandular area ↓ serum E2
2016	Lai	PCOS rats	EA	CV12, CV4, ST25	3 per wk for 5 weeks	↑ CPR, NBI	↑ INSR and IRS1 protein
2016	Lai	PCOS rats	EA	CV12, CV4, ST25	3 per wk for 5 weeks	↑ CPR, NBI	↑ IRS1 and IRS2 mRNA ↓ FINS and HOMA-IR
2022	Xing	PCOS rats	A	SP4, PC6, EX-CA1, CV4	Alternate day for 15 days	↑ NBI	↑ Endometrial morphology, pinopodes, HOXA10 and LIF protein expression, angiogenesis (PI3K/AKT pathway) ↑ VEGF, VEGFR2, Ang-1, PI3K, AKT, P-AKT gene/protein, eNOS and NO
2021	Xi	thin endometrium rats	EA	EX-CA1, SP6, CV4	Per day for 3 consecutive estrous cycles	↑ NBI	↑ EMT, glands and blood vessels numbers, pinopode-related markers ITG*α*v*β*3, HOXA10, HBEGF, ER*α*, PR
2021	Meng	thin endometrium rats	EA	CV4, EX-CA1, SP6	Per day for 10 days	NR	↑ Endometrial proliferation ER and PR proteins
2019	Xia	thin endometrium rats	EA	SP6, CV4, EX-CA1	Per day for three estrous cycles	↑ EIR	↑ SDF-1/CXCR4 axis, endometrial CK19 and vimentin, VEGF and bFGF
2023	Hao	thin endometrium rats	EA	CV4, SP9, EX-CA1	Per day for three estrous cycles	↑ EIR	↑ Endometrial glands number LIF proteins, CXCR4 proteins and mRNA
2016	Zhu	COH rats	EA	CV4, CV3, SP6	Per day till sampling	↑ CPR, NBI	↑ IGF-1 mRNA expression
2017	Dong	COH rats	A	ST36, SP6, LR3	Per day for 4 days	NR	↑endometrial angiogenesis ↑ VEGF, IL-15 and IL-18
2019	Chen	COH rats	EA	SP6, ST36, PC6, TE5	Per day for 3 days	↑ NBI	↑ endometrial angiogenesis VEGFR2/PI3K/AKT and VEGFR2/ERK axis
2020	Yuan	COH rats	A	BL26, SP6, BL23	Per day for 11 days	↑ NBI	↑ pinopodes, EMT, HOXA10 PI3K/Akt/mTOR pathway ↓ miR-494-3p
2021	You	COH rats	EA	CV4, ST36	For totally 31 days	↑ NBI	↑ EMT, LIF/STAT3 pathway ↓ E-cadherin, β-catenin and CLDN1
2022	You	COH rats	EA	CV4, ST36	For totally 31 days	↑ NBI	↓ E-cadherin, β-catenin and CLDN1, miR-223-3p expression ↑ LIF/STAT3 pathway
2023	Hu	COH rats	A	SP6, LR3, ST36	Per day for 7 times	↑CPR	↓ Serum P ↑ ER, PR, LIF, ITGβ3, VEGF and FGF-2
2024	Zhu	COH rats	EA	CV4, SP6	Per day till the end of modeling	NR	↑ EMT, glands and blood vessels numbers, HOXA10 and LIF mRNAs and proteins, BMAL1 mRNA ↓ Serum P
2007	Liu	EIF rats	A	ST36, SP6, LR3	Per day for 7 consecutive days	↑CPR, NBI	↑ Uterus weight
2014	Gao	EIF rats	A	ST36, SP6	Per day from D1 to death	↑ NBI	↑CXCR1 and CXCR2
2013	Gao	EIF rats	A	ST36, SP6	Per day from D1 to death	↑ NBI	↑ CCL2 and CXCL8 expression, subset of uNK cells
2012	Gui	EIF rats	A	ST36, SP6	Per day for 5–6 days	↑NBI	↑ LIF, IL-12 protein and mRNA expression
2012	Gui	EIF rats	A	ST36, SP6	Per day for 5–8 days	↑ NBI	↑ Th2 cytokines ↓ Th1 cytokines

ER, endometrial receptivity; PCOS, polycystic ovary syndrome; A, acupuncture; NBI, number of blastocyst implantation; EMT, endometrial thickness; T, testosterone; E2, estradiol; NR, not reported; CC, clomiphene citrate; LIF, leukemia inhibitory factor; OPN, osteopontin; EA, electropuncture; wk, Week; CPR, clinical pregnancy rate; INSR, insulin receptor; IRS, insulin receptor substrate; FINS, fasting insulin; HOMA-IR, homeostasis model assessment of insulin resistance; HOXA10, Homeobox A10; PI3K, Phosphatidylinositol-3-kinase; AKT, Protein kinase B; VEGF, vascular endothelial growth factor; VEGFR2, Vascular endothelial growth factor receptor2; Ang, Angiotensin; eNOS, endothelial nitric oxide synthase; NO, nitric oxide; ITG, integrin; HBEGF, Heparin-binding EGF-like growth factor; ERα, estrogen receptor alpha; PR, progesterone receptor; EIR, embryo implantation rate; SDF-1, Stromal cell-derived factor-1; CXCR4, C-X-C chemokine receptor type 4; CK19, Cytokeratin; bFGF, basic fibroblast growth factor; COH, controlled ovarian hyperstimulation; IGF-1, Insulin-like growth factor-1; IL, interleukin; ERK, Extracellular signal-regulated kinase; P, progesterone; FGF-2, Fibroblast growth factor 2; CLDN1, Claudin-1; STAT3, Signal transducer and activator of transcription 3; BMAL1, Brain muscle arnt-like 1; EIF, embryo implantation failure; CXCR1, CXC-chemokine receptor 1; CXCR2, CXC-chemokine receptor 2; CCL2, CC, chemokine subfamily L2; CXCL8, CXC, chemokine subfamily L8; uNK, uterine natural killer; Th2, T helper type 2; Th1, T helper type 1.

### Discussion on the potential mechanisms of acupuncture in enhancing endometrial receptivity

Impaired endometrial receptivity is one of the main causes of infertility in women, the sufficient evidence indicated that acupuncture could promote female fecundity by enhancing endometrial receptivity through multiple pathways. Based on the above-mentioned studies, we summarized and found acupuncture is helpful in improving endometrial morphology, regulating reproductive hormones, blood flow, and diverse cellular and immune molecules, which are commonly known indicators closely related to endometrial receptivity, therefore, acupuncture has a promising application value in infertility. Next, we discussed in detail the mechanisms by which acupuncture improves endometrial receptivity from the following five aspects.

### Improving endometrial morphology (including pinopodes, EMT and endometrial type a rate)

A well-developed endometrial morphology is crucial for embryo implantation. Pinopodes are smooth projections on the tips of the endometrial epithelium membrane, their development and degeneration are spatiotemporally synchronized with the opening and closing of the WOI. The number and shape of pinopodes are closely associated with the success rate of embryo implantation. They serve as a morphological indicator of optimal ER and as an ultrastructural marker of the WOI (Quinn et al., 2020). EMT is the most classic and widely used ultrasound-measured indicator for evaluating ER. The 2020 European Society of Human Reproduction and Embryology (ESHER) guidelines recommend that when the endometrium is ≥8 mm on the HCG trigger day or the transformation day, it is adequate for implantation (Ovarian Stimulation TEGGO et al., 2020). According to the Gonen and Capser classification criteria, the endometrial morphological on the HCG administration day could be categorized into three different types: Type A: a triple-line pattern, the outer and middle layers show strong echo, while the inner layer shows low echo, with distinct uterine midline echo; Type B: a relatively uniform high echo, same as the endometrial myometria image, with less obvious uterine midline echo; Types C: an entirely homogenous and strong echo, with no uterine midline echo (Gonen and Casper, 1990). Type A suggests the endometrium is in the proliferative stage, associated with a better clinical outcome, while type B and C imply the WOI may close early, leading to a low implantation rate (Gingold et al., 2015). Based on the reported studies above, we found most studies showed acupuncture could thicken the endometrium, raise the proportion of type A or type A + B endometrial pattern, and enhance the pinopodes expressions on endometrium, thus resulting in improved endometrial morphology and increased pregnancy outcomes.

### Enhancing the endometrial blood flow

Ultrasound-measured serial subendometrial and uterine artery fluxometry is a useful tool in predicting endometrial receptivity (Martins et al., 2019). The PI、RI and S/D are the three most frequently used indicators of helical arterial blood flow index to evaluate changes in the uterus after acupuncture treatment. Usually, a lower PI, RI and S/D are correlated with higher pregnancy rates. It is suggested endometrial blood flow provides a more direct reflection of the microenvironment at the embryo implantation site than uterine artery flow (Silva and Ginther, 2010). According to the Applebaum classification method, endometrial blood flow could be classified into three types: Type I: blood vessels crossed over the low-echo area of the lateral intima, but did not enter the outer edge of the hyperechoic intima; Type II: blood vessels crossed over the outer edge of the hyperechoic intima; Type III: blood vessels penetrate the intima (Applebaum, 1995). The Type III has richer blood flow compared to Types I and II, and is more conducive to increase pregnancy rate (Yang et al., 2024). Several studies (Shuai et al., 2015; Dong et al., 2024) evaluated the endometrial volume blood flow indices, covering the vascular index (VI), blood flow index (FI), and vascular blood flow index (VFI). Some novel ultrasound-related parameters, such as the endometrial vascular distribution index (VI^MV^ for endometrium) and bilateral ovarian vascular distribution index (VI^MV^ for ovary) are also being studied to evaluate the microvessels (Lai et al., 2024), which with slower blood flow velocity. In addition to ultrasound indicators, some serum markers, such as D-dimer, homocysteine (HCY) and thromboxane B2 (TXB2) were also detected to assess the prethrombotic state in RIF patients (You et al., 2023). The available literature supported the beneficial effect of acupuncture on endometrial blood flow. The evidence indicated that acupuncture could reduce uterine PI, RI and S/D and plasma D-dimer, HCY and TXB2 contents, increase type III of endometrial blood flow, elevate VI, FI and VFI, improve endometrial microcirculation, so as to promote the blood supply to the endometrium and enhance the endometrial tolerance.

### Bidirectionally adjusting levels of E2 and P and their receptors

Physiologically, the endometrium undergoes periodically changes, including shedding, regeneration and differentiation in a menstrual cycle, which are primarily driven by the mutual coordination of E2 and P signaling pathways (Junhan et al., 2023). During the proliferative phase, the E2 predominates, it mainly binds to receptor α to stimulate the proliferation of epithelial and stromal cells (Marquardt et al., 2019). After ovulation, the elevated P level via its receptor induces the endometrium transforms into a secretory phase, in which the stromal cells undergo decidualization, so as to provide optimal environment for embryo implantation and pregnancy (Marquardt et al., 2019). Imbalances or disorders in E2 and P signaling not only influence the oocytes quality, but also lead to decreased endometrial receptivity, thus affecting fertility (Günther et al., 2023). We found that acupuncture may exert a bidirectional regulatory effect on hormone levels. For example, in PCOS patients entering IVF-ET cycle or COH rats model, large dosage of exogenous ovulation-inducing drugs could produce superphysiological levels of serum E2 on the HCG injection day, which will induce ovary hyperstimulation syndrome and disrupt the endometrial development process (Nastri et al., 2010). In addition, an early increase in P levels on the HCG trigger day could make the WOI forward and lead to asynchrony between the embryo and endometrium, thereby reducing the pregnancy rate (Labarta et al., 2011). Acupuncture could reduce the elevated serum E2 and P levels, so as to achieve an appropriate hormone state for subsequent embryo implantation (Wu et al., 2022; Hu et al., 2023; Zhu et al., 2024). While in patients undergoing IVF-ET treatment for fallopian tube factors (Chen and Hau, 2015) or PCOS patients (Yu et al., 2018), or thin endometrium type rats (Xi et al., 2021; Meng et al., 2021), acupuncture showed enhanced effects on the E2 and P levels and their receptors, so as to promote endometrial proliferation and thickening, enhance the luteal support, and improve the endometrial receptivity during the WOI.

### Regulating molecular biological factors

E2 and P signaling pathways activate a series of downstream target molecules to guide the structural and functional remodeling in the endometrium, where multiple factors related to uterine receptivity are expressed. LIF is the first and most lasting endometrial protein essential for embryo implantation (Cullinan et al., 1996; Wang and Wang, 2022), which is continuously and dynamically expressed throughout the menstrual cycle, and significantly increases from the mid-secretory to late-secretory phase. LIF is considered as a key factor in embryo implantation (Arici et al., 1995; Vogiagis et al., 1996). HOXA10, as a homeobox-containing transcriptional factor, plays a pivotal role in promoting the endometrial decidualization by regulating genes essential for implantation (Bagot et al., 2001; Das, 2010). Integrin β3, an essential molecule involved in the embryo’s initial attachment and adhesion, can facilitate trophoblast invasion and subsequent implantation (Cai et al., 2023). During embryo implantation, angiogenesis is a critical process determines the ER and the opening of WOI. VEGF serves as the regulatory center for angiogenesis and is regarded as the representative biomarker of angiogenesis (Karizbodagh et al., 2017). IGF-1 a member of the IGF family, it was found that higher content of IGF-1 during WOI indicated better embryo implantation ability (Chang et al., 2022). These molecular markers are highly expressed in the middle and late luteal periods and coincide with the WOI. While, in infertile patients, their abnormal decrease in expressions during WOI are associated with poor ER and lead to implantation failure. Given the findings, acupuncture may enhance endometrial receptivity by improving these factors through activating the specific signaling pathways to make the implantation window return to normal condition.

### Modulating endometrial immune-inflammatory microenvironment

During implantation, the endometrium creates an immune-tolerant microenvironment at the maternal-fetal interface, to prevent the embryo, a semillgenicgraft, from being attacked by the mother, thereby helping to establish and support pregnancy (Murata et al., 2021). Uterine immune cells are, therefore, vital in the regulation of endometrial receptivity. uNK cells are the most prevalent innate lymphocyte population, which are responsible for regulating trophoblast invasion and the maternal vascular development and remodeling by interacting with other immune cells (Male and Moffett, 2023). Treg cells are another kind of decidual leukocytes, which accounts for smaller proportion than uNK cells. Treg cells can be divided into two different subsets based on their function: “the bad” and “the good”: Th1 cells are one type of pro-inflammatory cells, they stimulate the secretion of TNFα, IL-1, IL-2, and IFNγ, which are detrimental to implantation; while the Th2 cells demonstrate anti-inflammatory effect, their predominant production, such as IL-4 and IL-10 are beneficial to implantation and pregnancy (Robertson et al., 2022). In addition, chemokines are a large superfamily of cytokines, represented by CXCL8 and CCL2, these chemokines play important role in successful implantation by the chemotaxis of leukocytes, inducing trophoblast migration, modulating cell proliferation and promoting adhesion (Złotkowska and Andronowska, 2019). Clinical and animal studies have shown women with RIF or recurrent miscarriage may present as alterations in abundances or functions of these immune cells (Nupur et al., 2023), which poses a potential target for clinical treatment. Based on the reported studies, we can find acupuncture could upregulate the CCL2 and CXCL8 expressions, improve the subset of uNK cells, balance the Th2/Th1 ratio and decrease adhesion molecules of E-cadherin, β-catenin and CLDN1 expressions at the maternal-fetal boundary, thus modulating the immune-inflammatory microenviroment and contributing to embryo implantation.

### Safety of acupuncture in female infertility

There are very few reports on the adverse events associated with acupuncture treatment in female infertility. A systematic review found that the incidence of adverse events in the acupuncture group was significantly higher than the control group. However, the rate was lower in the sham acupuncture group compared to the true acupuncture group. Despite this, the finding suggested that both sham and true acupunctures are comparably safe and effective (Quan et al., 2022). Another systematic review and meta-analysis (Zhong et al., 2019) on acupuncture’s effect in improving ER, which included 13 RCTs on women with infertility due to low ER, reported adverse events in only 3 studies. Only one case of fainting during acupuncture treatment was reported in the experimental group [164]; one study reported 3 cases of gastrointestinal indigestion in the control group, whereas no adverse events were observed in the experimental group (Yu et al., 2018); another study noted that neither the experimental group nor the control group experienced adverse events (Qu et al., 2017). Since all of these clinical studies had small sample size and low evidence, additional large-scale clinical trials were needed before conclusions reached. A newly published large-scale RCT reported adverse events in 152 women undergoing IVF (Smith et al., 2018). All events were minor and specific to acupuncture such as discomfort and bruising, with a statistically significantly higher incidence of discomfort in the acupuncture group. Reported adverse events associated with acupuncture were extremely limited and generally less severe compared to other control groups. These events can largely be prevented through careful and hygienic administration, as well as proper patient education.

## Summary and controversy

An excellent endometrium serves as the final barrier to achieving a successful pregnancy. Among female infertility cases, the poor endometrial receptivity accounts for a large proportion and the etiology is multifactorial. This review discusses the common pathological causes of endometrial receptivity defects, including PCOS, thin endometrium, uterine abnormalities, chronic endometritis, immune-mediated disorders, etc., which is envisaged to help target the clinical diagnosis and treatment for improving endometrial receptivity.

Acupuncture, a complementary and alternative therapy, has been practice for thousands of years. Base on the reported researches, acupuncture demonstrates a beneficial effect in treating female infertility, with significant clinical application value of improving endometrial receptivity. The present study reviewed the feasibility and efficacy of acupuncture for improving pregnancy outcomes and summarized the potential mechanisms of enhancing endometrial receptivity may act through the following five pathways, including: improving endometrial morphology, increasing the endometrial blood flow, bdirectionally adjusting levels of E2 and P as well as their receptors, regulating molecular biological factors beneficial for implantation, and modulating endometrial immune-inflammatory microenvironment at the maternal-fetal interface, etc. To the best of our knowledge, this is the most comprehensive review to evaluate the acupuncture-specific action and mechanisms on endometrium from both clinical and animal reports.

In clinic, acupuncture has been widely used as an auxiliary therapy to improve the effectiveness of treatments for infertility. In addition to its therapeutic effect, the advantages of acupuncture include its simple operation and safety profile. And fortunately, the medical insurance fund had reimbursed for the technology in China, which means the expense only a few dozen yuan per time, hence, it is considered relatively cost-effective.

However, some limitations should be proposed. At present, the results of current researches are not completely consistent. We suppose it may be due to large heterogeneity among study designs (including infertility type, sample size, intervention mode and timing, treatment course, acupoint selection, stimulation frequency and intensity, the angle and depth of acupuncture, etc.), and primary reported outcomes (ovulation rate, embryo implantation rate, clinical pregnancy rate, live birth rate, miscarriage rate), resulting in less generalized conclusions in these studies. In addition, currently, the depth and breadth of action mechanisms of acupuncture on infertility and endometrial receptivity are still not sufficient. In recent years, the modern medicine reveals the effect of acupuncture stimulation can be realized by activating the somatosensory-autonomic reflex pathway. From the perspective of neuroanatomy, the acupoints are mostly regions rich in collagen fibers, where there are abundant sensory nerves. In clinical applications, acupoints Zigong (EX-CA1), Guanyuan (CV4), Sanyinjiao (SP6), Ciliao (BL32) and Neiguan (PC6) are the widely used acupoints in infertility treatment. Among them, EX-CA1 is located at the T12 segment (Tang et al., 2020); CV4 is located at L1∼L2 segments, BL32 is located at S2 segment, SP6 are located at the tibial nerve innervation region, and PC6 is the acupoint has been proven to affect vagus nerve (Chang et al., 2023). The uterus is mainly innervated by the sympathetic and parasympathetic nerves (Baljet and Drukker, 1980). The sympathetic pre-ganglionic fibers come from the T11-L2 spinal cord segments, which is responsible for uterine and vascular contractions (Wall et al., 1993); while the parasympathetic pre-ganglionic fibers come from the S2-S4 spinal cord segments, and mainly induce uterine and vascular dilation (Berkley et al., 1993). In addition, the tibial nerve, originates from the L4-S3 segment, also emits nerves to innervate the pelvic floor. Thus, it can be seen that these main points for infertility treatment are anatomically correspond with the innervation of the uterus, which indicates that acupuncture may regulate the functions of the uterus by stimulating the somatosensory-autonomic nervous system, namely the sacral nerve, vagus nerve and tibial nerve. Indeed, neural regulation technology has been applied in the medical field since the 1970s and has gradually gained widespread recognition, including vagus nerve stimulation (VNS), sacral nerve modulation (SNM) and tibial nerve stimulation (TNS), all of which have been approved by the FDA. Fortunately, we have previously done some work on the VNS and firstly proposed the non-invasive percutaneous electrical VNS could be a novel treatment for PCOS infertility, which has been published elsewhere (Shike et al., 2023). Uterine autonomic nerve dysfunction has been demonstrated to impair fertility (Newman et al., 2013). According to the latest anatomical study by Pinsard et al., the distribution of uterine nerves has been systematically described, confirming the dominant role of sympathetic nerves in the endometrium and arteries (Pinsard et al., 2022). Based on the anatomical evidence that sympathetic innervation of the endometrium originates from the T12-L2 spinal segments, we propose an acupuncture strategy targeting the corresponding acupoints at these segments: Jiaji (EX-B2) or Back-Shu points (BL21-BL23). This therapeutic approach aligns with the Traditional Chinese Medicine concept of “Qijie”, which emphasizes the functional correlation between spinal segments, visceral innervation, and the efficacy of acupoints. However, whether these techniques act through stimulating the relevant nerves innervating the uterus to regulate endometrial receptivity still lacks direct researches, currently.

To summarize, the present study not only summarizes the efficacy and mechanism of acupuncture for ER in female infertility but also provides meaningful guidance for clinic application as well. Future studies should include high-quality, large-sample, double-blind RCTs to enhance the level of evidence. Additionally, with the development of modern medicine, it is increasingly believed that neural reflex is fundamental in producing the clinical effects of the acupoints, future the basic experiments could be designed from the view of neuroendocrine to clarify the mechanism of acupuncture in improving endometrial receptivity in female infertility.

## Conclusion

**FIGURE 1:**
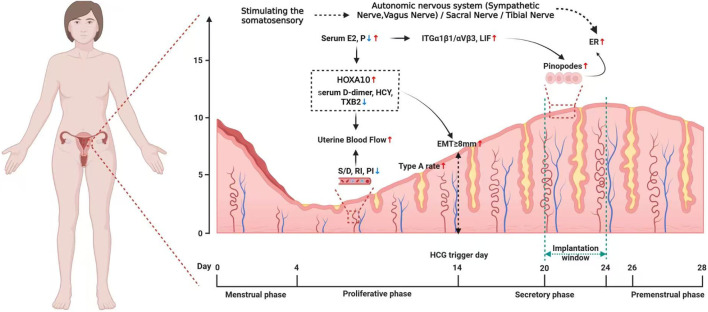
Potential mechanisms by which acupuncture enhances endometrial receptivity in women.

**FIGURE 2:**
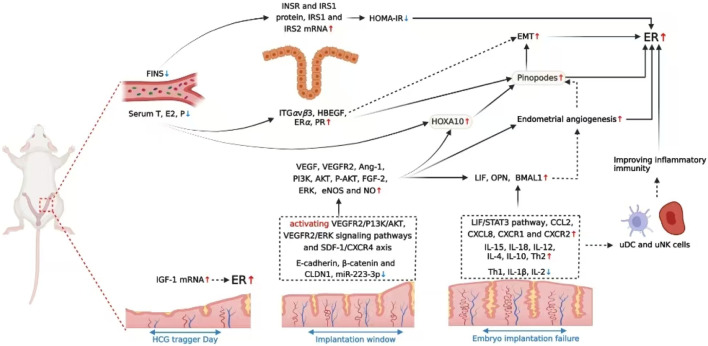
Potential mechanisms by which acupuncture enhances endometrial receptivity in animals.

Overall, the review suggested acupuncture treatment could improve pregnancy outcomes by increasing endometrial receptivity, which may act through multiple pathways including improving endometrial morphology, increasing the endometrial blood flow, bdirectionally adjusting levels of E2 and P as well as their receptors, regulating molecular biological factors, modulating endometrial immune-inflammatory microenvironment, and probably activating the somatosensory-autonomic reflex pathway, etc. (as shown in Figures 1,2 ). Thus, acupuncture is a convenient, safe and economical manipulation and could be used as an adjunct therapy for enhancing endometrial receptivity in PCOS, thin endometrium, DOR and ART related infertility, with significant clinical application value.
